# A method for manual and automated multiplex RNAscope *in situ* hybridization and immunocytochemistry on cytospin samples

**DOI:** 10.1371/journal.pone.0207619

**Published:** 2018-11-20

**Authors:** Sara Chan, Audrey Filézac de L’Etang, Linda Rangell, Patrick Caplazi, John B. Lowe, Valentina Romeo

**Affiliations:** 1 ISH/IHC core—Department of Pathology, Genentech Inc., South San Francisco, California, United States of America; 2 Department of Neuroscience, Genentech Inc., South San Francisco, California, United States of America; 3 Department of Research Pathology, Genentech Inc., South San Francisco, CA, United States of America; Western College of Veterinary Medicine, University of Saskatchewan, CANADA

## Abstract

*In situ* analysis of biomarkers is essential for clinical diagnosis and research purposes. The increasing need to understand the molecular signature of pathologies has led to the blooming of ultrasensitive and multiplexable techniques that combine *in situ* hybridization (ISH) and immunohistochemistry or immunocytochemistry (IHC or ICC). Most protocols are tailored to formalin-fixed paraffin embedded (FFPE) tissue sections. However, methods to perform such assays on non-adherent cell samples, such as patient blood-derived PBMCs, rare tumor samples, effusions or other body fluids, dissociated or sorted cells, are limited. Typically, a laboratory would need to invest a significant amount of time and resources to establish one such assay. Here, we describe a method that combines ultrasensitive RNAscope-ISH with ICC on cytospin cell preparations. This method allows automated, sensitive, multiplex ISH-ICC on small numbers of non-adherent cells. We provide guidelines for both chromogenic and fluorescent ISH/ICC combinations that can be performed either in fully automated or in manual settings. By using a CD8+ T cells *in vitro* stimulation paradigm, we demonstrate that this protocol is sensitive enough to detect subtle differences in gene expression and compares well to commonly used methods such as RT-qPCR and flow cytometry with the added benefit of visualization at the cellular level.

## Introduction

Analysis of biomarkers, such as nucleic acids and proteins, is essential for clinical diagnostic and basic research purposes [1]. *In situ* hybridization (ISH) and immunohistochemistry (IHC) are the methods of preference to determine *in situ* biomarkers expression. Both techniques offer a semi-quantitative identification of target nucleic acids or proteins, by conserving topological information of expression within cells and with respect to the surrounding structures. This information is in fact lost with other detection methods, such as single cell RNAseq, qPCR, western blotting or flow cytometry, for which tissues must be dissociated. Other limitations, especially of qPCR and western blotting are (i) the averaging of signal detection due to usage of bulk tissues and (ii) the necessity to identify a suitable normalizer, that is often a non-trivial issue [2].

RNA *in situ* hybridization (RNA ISH) has been a valuable tool for investigating mechanisms of cellular pathology since the 1970s. Through prior decades, RNA ISH underwent a series of improvements including increased safety, protocol simplification, increased robustness and sensitivity [3]. Currently, multiple approaches exist to carry out RNA ISH [4, 5]. Among them, RNAscope technology excels for robustness and sensitivity. It takes advantage of a variation of the branched DNA or “tree” amplification method: multiple tandem probes hybridized to the target transcript are pre-amplified by an adapter, to which multiple amplifier probes are then attached. In a final step, detection probes are bound to the amplifiers and signal is detected. RNAscope offers several detection possibilities for both chromogenic and fluorescent assays. The usage of multiple tandem probe-sets in the same assay directed to the same target mRNA increases the chance of detecting low expressed or even partially degraded targets. This, together with the multiple amplification steps, yields unprecedented sensitivity. Further, the pre-amplification assures increased specificity through the following amplification steps, beyond direct probe-to-target base pairing. In fact, the pre-amplifier molecule can only bind two probes that are associated with the target mRNA in tandem, thus dramatically reducing the chances that an off-target hybridization event lead to non-specific signal amplification. RNAscope signal is revealed as a punctate staining, with little or no background, and previous studies demonstrated that each punctum corresponds to one molecule of the intended target mRNA [6, 7]. Thus, quantification of the number of puncta per cell offers a direct measurement of expression of a certain target. However, for highly expressed genes, puncta can oftentimes overlap and fuse, resulting in a difficult accurate numeric quantification. A solution to this issue may be to quantify the median signal intensity (MFI) of a certain signal within a cell. RNA ISH is a powerful tool to detect mRNA expression; nevertheless, mRNA levels not always directly correlate with respective protein expression due to post-transcriptional regulation mechanisms [8]. In addition to the features described above, which, in essence provide exquisite sensitivity, specificity and robustness, RNAscope uses relatively gentle chemistry for tissue permeabilization as well as low temperatures, rendering the protocols uniquely suitable for following detection of proteins. In fact, identical buffers can be used for RNAscope ISH or epitope retrieval in immunocytochemistry.

Immunohistochemistry or immunocytochemistry (ICC) allow visualization of protein antigens within tissues and cells. These methods are very well established and widely used in many fields of research [9, 10]. However, IHC and ICC performance depends heavily on the existence of an antibody that binds to the intended target with a suitably strong affinity, in addition to other factors. Despite their respective limitations, ISH and IHC remain the gold standards for *in situ* analysis of biomarkers. Combination of those methods can overcome the limitations inherent in a single ISH or IHC/ICC assay.

Historically, the analysis of multiple markers was performed on consecutive tissue sections, thus loosing information about co-localization within the same cell population. Multiplexing histochemistry is necessary to overcome the limitations of studying serial tissue sections and to better understand molecular signatures of pathologies [11]. Current technologies utilize fluorescence or mass spectrometry for accurate detection of several, typically protein, analytes. In addition, significant efforts have been made to achieve quantitative objective evaluation of biomarker expression in high-throughput settings [11, 12]. Consequently, staining automation has become an integral part of routine pipelines, to support robustness and reproducibility, together with output increment.

Generic protocols are available to combine ISH assays and IHC [13, 14]. However, these protocols are mostly tailored for formalin-fixed paraffin-embedded (FFPE) or frozen tissue sections. Commonly, non-adherent cells are processed for ISH/IHC by generation of cell pellets that are treated as tissue blocks. These processes require a high number of cells and extensive handling. There are cases in which samples are limited, such as for a single patient blood-derived PBMCs, rare tumor samples (e.g. circulating tumor cells), effusions or other body fluids, dissociated or sorted cells. Cytospin is an excellent tool to overcome this limitation [15]. Cytospin is a centrifugation-based method that concentrates samples with low cellularity on a glass slide that is suitable for microscopy. Importantly, cells processed by cytospin maintain natural cell morphology, thus allowing evaluation of pathological changes, and can tolerate downstream processing by ISH and ICC [15, 16].

For this study, we investigated mouse primary naïve CD8+ T cells activation *ex vivo*. Naïve CD8+ T lymphocytes are a relatively rare population (0.4–2.6% of total leukocytes in human peripheral blood and about 12% of total cells in mouse spleen) of small cells, characterized by a heterochromatic nucleus, and a ring of cytoplasm that consist of about 10–20% of the entire cell volume [17, 18]. Upon activation, CD8+ T cells undergo a number of molecular changes that result in expression of activation markers that also have a role in sustaining effector functions (e.g. CD69, CD25) and the downregulation of naïve cells markers (e.g. CD62L) [19–22]. Activated CD8+ T cells also undertake extensive morphology remodeling, resulting in cytoplasm enlargement and chromatin decondensation, both clearly visible within 24 hours of stimulation *in vitro*.

Here we describe a method that combines ultrasensitive RNAscope-ISH with ICC on cytospin cell preparations. This method allows automated, sensitive, multiplex ISH/ICC on small numbers of non-adherent cells. With the aim to provide guidelines for multiple uses and set the bases for further customization, we outline protocols for both chromogenic and fluorescent ISH/ICC combinations that can be performed either in a fully automated or in a manual setting. The sensitivity of the described methods is high enough to detect and quantify subtle variation in expression kinetics of endogenous targets, with specificity comparable to other high-sensitivity methods such as RT-qPCR and flow cytometry.

## Material and methods

### Animals

Adult 12–16 weeks old C57BL/6J mice, obtained from Jackson Laboratory, were used for these experiments. All animal studies, animal care, housing, ethical usage and procedures were performed in accordance with standard regulations and were approved by Genentech Institutional Animal Care and Use Committee (IACUC). Mice were group housed in IVC cages (3–5 mice per cage) on a 12 hours light/dark cycle with food and water available *ad libitum*. Animals were euthanized by CO_2_ inhalation. Spleen and lymph nodes were harvested according to IACUC protocol# TH17-0056. For each experiment 8–12 animals were euthanized in order to obtain enough cells to perform the various methods optimizations. A total of 60 animals were used in these studies.

### Primary naïve CD8+ T lymphocytes isolation and stimulation

Spleen and lymph nodes were collected in GentleMACS C-tubes (cat # 130-096-334) containing 5 mL complete medium (RPMI 1640 with Glutamax- Gibco cat # 61870–036, 10% heat inactivate FBS- hyClone cat # SH3008803HI, 1mM sodium pyruvate -Gibco cat # 1136–070, 55uM b-mercaptoethanol- Gibco cat # 21985–023, 10mM HEPES pH 7.2). Tissues were homogenized with a GentleMACS OCTO dissociator. CD8+ T lymphocyte isolation was performed with Dynabeads untouched mouse CD8 cells kit (cat # 11417D, Invitrogen), according to the manufacturer’s protocol. Isolated cells were counted and resuspended at 2x10^6^/mL in complete medium. 4x10^6^ cells were plated in each well of 6 well plates pre-coated with anti-CD3e antibody (BD Pharmingen purified NA/LE hamster anti-mouse CD3e clone 145-2C11, cat # 553057) at 10ug/mL, or non-coated plates for control, and incubated at 37°C for 1, 2, 3, 4, 24 hours. Non-stimulated (Naïve) samples were kept on ice until fixation.

### Human primary monocyte-derived dendritic cells

Primary monocytes were purified from buffy coats with monocyte isolation kit II (MACS cat # 130-091-153), accordingly to manufacturer instructions. Cells were treated for 5 days in complete medium (RPMI 1640 with Glutamax- Gibco cat # 61870–036, 10% heat inactivate FBS- hyClone cat # SH3008803HI, 1mM sodium pyruvate -Gibco cat # 1136–070, 55uM b-mercaptoethanol- Gibco cat # 21985–023, 10mM HEPES pH 7.2) complemented with 800 U/ml of GM-CSF (peprotech cat # 300–03) and 500 U/mL IL-4 (peprotech cat # 200–04).

### Cytospin

RNAscope Technical Note for Non-Adherent cells protocol (Advanced Cell Diagnostics, Newark, CA, ACD, technical note 321230 RevA) was used for fixation, post-fixation, wash and storage steps. Purified lymphocytes were centrifuged at room temperature (RT) at 250 RCF for 10 minutes in 50 mL polypropylene tube. Supernatants were removed and cells were washed with 10 mL PBMC buffer (ACD, 320972). Samples were then centrifuged at 250 RCF for additional 10 minutes. Supernatants were removed and cells were resuspended in 5 mL of 10% neutral buffered formalin (NBF) by gently pipetting 10 times to break apart the cell pellet and incubated at 37°C for 30 minutes. Fixed samples were centrifuged at 250 RCF for 10 minutes and supernatants were removed. Cells were resuspended in a volume of 70% Ethanol to adjust the cell density to 1x10^6^ cells per mL, to produce a monolayer on a slide. Samples were centrifuged at 800 RCF for 10 minutes and the slides were removed from cytoprep kit. Slides were air dried for 20 minutes at RT and dehydrated in 50%, 70% and 100% Ethanol in preparation for staining.

### Manual procedures

Manual cytospin ISH is a modified protocol of pretreatment steps from Advanced Cell Diagnostics (ACD, 322360-USM) adjusted for cytospin samples; subsequent hybridization and amplification steps remained as in the original protocol. Fast Red from ScyTek (FR0001) was used for ISH signal detection instead of the Fast Red provided by ACD due to a better compatibility with Tissue Tek mounting medium, that also was preferred to Ecomount.

#### 1. Sample pretreatment

After cytospin, slides were removed from 100% EtOH and dried for 30 minutes in an oven at 37°C. A hydrophobic barrier was created around the cell spot with a hydrophobic barrier pen, and air dried for 1 minute. Enough 3% hydrogen peroxide solution (H_2_O_2_) was added to cover the sample and incubated for 10 minutes at RT. After incubation, H_2_O_2_ was removed and slides were washed twice with distilled water. Protease plus (ACD, 322330) was added to the samples and incubated in a HybEZ oven for 30 minutes at 40°C. After incubation, protease was removed by submerging the slides in 1X PBS buffer for 2X 1 minute each.

#### 2. Probe hybridization and amplification

Hybridization and amplification steps were done according to the RNAscope protocol provided by ACD. Probes were hybridized for 2 hours at 40°C in a HybEZ oven. Slides were washed with 1X wash buffer 2 times 2 minutes each at RT following each amplification step using RNAscope 2.5 HD Reagents Detection Kit-RED (ACD, 32360).

#### 3. ISH signal detection

Fast Red (ScyTek, FR0001) reagent was prepared with Napthol Phosphate Buffer according to manufacturer’s instructions. Fast Red mixture was applied to the tissue section on the slide and incubated for 5 minutes. Tissue samples were rinsed with deionized (DI) water. A second round of Fast Red mixture was applied to the tissue section and incubated for additional 5 minutes. Tissue samples were rinsed with DI water 5 times. Slides were washed in ACD wash buffer for 1 minute in preparation for the Immunocytochemistry (ICC) procedure. Probes used in this study are listed below (Table 1).

**Table 1:** RNAscope probes used in this study.

Probe Names	Catalog Number	Opal Fluor	Reference Number
RNAscope 2.5 LS Mm-Ppib	313918	Opal 570 Red	Perkin Elmer FP1488001KT
RNAscope 2.5 LS DapB	312038	Opal 570 Red	Perkin Elmer FP1488001KT
RNAscope 2.5 LS Mm-Cd69	449338	Opal 570 Red	Perkin Elmer FP1488001KT
RNAscope 2.5 LS Mm-Cd69-scrambled	518168	Opal 570 Red	Perkin Elmer FP1488001KT
RNAscope 2.5 LS Mm-Notch1	404648	Opal 570 Red	Perkin Elmer FP1488001KT
RNAscope 2.5 LS Mm-Notch1-scrambled	511698	Opal 570 Red	Perkin Elmer FP1488001KT
RNAscope 2.5 LS Mm-Notch1-C2 (for multiplex)	404648-C2	Opal 620 Purple	Perkin Elmer FP1495001KT
RNAscope 2.5 LS Mm-Notch1-scrambled-C2 (for multiplex)	511698-C2	Opal 620 Purple	Perkin Elmer FP1495001KT
RNAscope 2.5 LS Hs-ITGAX (aka CD11c)	419158	Opal 620	Perkin Elmer FP1495001KT

#### 4. Manual immunocytochemistry

Endogenous peroxidase activity was quenched with 3% hydrogen peroxide (H_2_O_2_) (Sigma-Aldrich, H1009) in 1X PBS for 4 minutes at RT and washed with TBST (0.05M Tris, 0.15M NaCl, 0.05% Tween-20) for 30 minutes at RT. Secondary antibody species specific serum was used to block for 30 minutes at RT. Sera were removed and slides incubated with primary antibodies at RT for 60 minutes. Slides were then washed with TBST 3 times and incubated with secondary antibodies for 30 minutes at RT. Slides were then washed with TBST 3 times and the reaction was visualized using Vina-Green for 5–10 minutes at RT (BioCare, BRR807). Vina-Green solution was prepared according to the manufacturer’s directions. Samples were washed with DI water and lightly counterstained with hematoxylin and bluing. Excess bluing was rinsed off with DI water 2 times 2 minutes each. The slides were dried in 55°C oven for 30 minutes or until dried. Slides were mounted with Tissue Tek Mounting Medium (Sakura, cat # 6419) xylene-based permanent mounting medium.

### Automated procedures

Automated cytospin ISH is a modified single ISH protocol from Advanced Cell Diagnostics RNAscope 2.5 LS Reagent Kit-Red User Manual (ACD, UM-322150 RevA), performed using a Leica Bond-RX system. Pretreatment steps were adjusted to maintain optimal morphology for cytospin samples. Sample pretreatment and probe hybridization steps were the same for both chromogenic and fluorescent procedures, while fluorescent ISH procedure was modified from ACD protocol in the amplification steps.

#### 1. Sample pretreatment

Pretreatment steps for all single, dual and multiplex cytospin assay development followed the same procedure. Slides were removed from 100% EtOH and dried in an oven at 37°C for 30 minutes. Slides were labeled with ACD2.5 Red Rev B protocol (without counterstaining step if dual or multiplex procedure) and inserted into the Bond RX slide racks to be processed. The existing software defined protocol steps (designed for single label immunohistochemistry) were modified or “repurposed” in order to account for the sample type and desired run conditions. Accordingly, pre-existing “pretreatment” step was replaced with “frozen slide delay” step to accommodate the overnight delay run. Antigen retrieval was conducted with *ACD HIER 15 minutes with ER2 at 88°C (Bond Epitope Retrieval Solution 2; Leica Cat # AR9640). Enzyme digestion step was omitted to avoid over-digestion of the sample. Please note that automated procedure is not precluded to epitopes that require protease digestion. Protease digestion step must be optimized for the cytological sample of choice. Endogenous peroxidase activity was quenched with RNAscope 2.5 LS hydrogen peroxide for 10 minutes at RT and washed twice with 1X Bond wash buffer (Leica 10X concentrate Cat # AR9590). Peroxide quenching step was re-added to the hybridization protocol as a work-around because enzyme treatment and quench step were linked in the automated program. Therefore, removal of the enzyme treatment also leads to the removal of the quenching step. This work-around is not necessary if enzyme digestion step is not eliminated.

#### 2.a_Chromogenic single *in situ* procedure

Following sample pretreatment, hybridization and amplification steps were done according to the RNAscope LS2.5 protocol (ACD, UM-322150 RevA). Probes were hybridized for 2 hours at 42°C. Slides were washed with 1X Bond wash buffer (Leica 10X concentrate, AR9590) at 42ºC 3 times (0, 1, 5 minutes) followed by 8 washes with 1X Bond wash buffer 0 minute each. Slides were then treated with Amp 1 to Amp 6 steps using RNAscope 2.5 LS Reagents Kit-Brown (ACD, 322100).

ISH detection and counterstain were completed using Bond Polymer Refine kit (Leica, DS9800).

#### 2.b_Chromogenic dual ISH/ICC

For dual ISH/ICC, counterstaining was omitted to include ICC. ISH detection was performed as described above.

Upon completion of ISH detection, slides were again treated with RNAscope 2.5 LS Hydrogen Peroxide to quench endogenous peroxidase for 10 minutes at RT and 3 washes with 1X Bond wash buffer. Slides were treated with species specific serum for 30 minutes at RT to block non-specific binding, prior to incubation with primary antibody for 60 minutes at RT. After primary antibody incubation, slides were open washed 3 times with 1X Bond wash buffer. HRP-conjugated secondary antibody was added for 30 minutes at RT, then 6 open washes with 1X Bond wash solution were performed. Final detection was done using Vina-Green for 5–10 minutes at RT (BioCare, BRR807). Vina-Green solution was prepared according to the manufacturer’s directions. Samples were washed with DI water and lightly counter stained with Hematoxylin and Bluing. Excess bluing was rinsed off with 2 washes in DI water for 2 minutes each. The slides were dried at 55°C for 30 minutes or until dried. Slides were mounted with Tissue Tek Mounting Medium (Sakura, cat # 6419) xylene-based permanent mounting medium.

#### 2.c_Fluorescent single ISH procedure

Fluorescent cytospin single ISH is a modified staining protocol of single chromogenic RNAscope 2.5 LS Red detection (322150-USM) using RNAscope 2.5 LS reagent kit (ACD, 322150). Sample pretreatment steps were the same as chromogenic single ISH.

Following sample pretreatment, probe hybridization and wash steps outlined in the single chromogenic ISH procedure, samples were processed only to the end of the Amplification 4 step (*ACD Amp4) followed by washes.

ISH detection was performed using Opal-570 (1:1500) in 1X amplification buffer (PerkinElmer, NEL794001KT) incubated for 1 and 10 minutes each at RT. Slides were washed with 1X Bond wash solution 3 times 0 minute (i.e. by immediately replacing the wash solution) each followed by additional 5 times 1 minute each at RT. Slides were counterstained with spectral DAPI for 1 and 5 minutes at RT. Excess DAPI was rinsed off by 4 washes with DI water. Finally, the slides were cover slipped with Prolong Gold anti-fade reagent (Life Technology Cat # P36930).

#### 2.d_Fluorescent dual ISH/ICC

Following probe hybridization and wash steps outlined in the single chromogenic ISH procedure, samples were processed only to the end of the Amplification 4 step (*ACD Amp4) followed by washes.

ISH detection was completed using Opal-570 (1:1500) in 1X amplification buffer (PerkinElmer, NEL794001KT) 1 and 10 minutes each at RT. Slides were washed with 1X Bond wash solution 3 times 10 minutes each followed by additional 5 times 1 minute each at RT. Slides were then rinsed 2 times with DI water and continued to ICC procedure.

Upon completion of ISH detection, slides were again treated with RNAscope 2.5 LS Hydrogen Peroxide to quench endogenous peroxidase for 10 minutes at RT and 3 washes with 1X Bond wash buffer. Slides were incubated with TNB blocking (0.1M Tris-HCl, pH7.5, 0.15M NaCl, 0.5% Blocking Reagent PerkinElmer, FP1012) for 30 minutes at RT. Primary antibody was incubated 60 minutes at RT. Slides were then open washed 3 times with 1X Bond wash buffer. HRP-conjugated secondary antibody was added for 30 minutes at RT, and then 6 open washes with 1X Bond wash solution were performed. Final detection step was conducted with PerkinElmer Opal dye (1:1500) in 1X amplification buffer incubated for 30 minutes at RT. Excess dye was removed with 8 open washes with Bond wash solution. Spectral DAPI (PerkinElmer. FP1490) counterstain was performed for 5 minutes at RT. Excess DAPI was rinsed off by 5 washes with DI water. Finally, the slides were cover slipped with Prolong Gold anti-fade reagent (Life Technology Cat # P36930).

#### 2.e_Fluorescent multiplex ISH/ICC procedure

Dual fluorescent ISH for multiplex was a modified RNAscope LS Multiplex Fluorescent protocol from Advanced Cell Diagnostics (ACD, 322800-USM) using RNAscope LS Multiplex Fluorescent Reagent Kit (ACD, 322440). Upon completion of pretreatment steps, slides were subjected to probe hybridization, followed by washes. Samples were processed through the completion of ACD Multiplex TSA-F2 development and washed again. Upon completion of ISH detection, slides were treated with ACD Multiplex HRP blocker for 10 minutes to ensure that all the excess HRP were quenched, followed by 3X washes with 1X Bond wash buffer and continue to ICC procedure. After quenching, slides were incubated with TNB blocking for 30 minutes at RT. Cocktailed primary antibodies were incubated simultaneously for 60 minutes at RT. Slides were open washed 3 times with 1X Bond wash buffer, subsequently incubated with Goat anti-Rabbit-HRP (1:1000) (PerkinElmer, Cat # NEF812001EA) for 30 minutes at RT and open wash 6 times with 1X Bond wash solution. Final detection of anti-Notch1 ICC was conducted with PerkinElmer Opal-520 (1:1500) in 1X amplification buffer, incubated for 30 minutes at RT. Excess dye was removed by washing 8 times with 1X Bond wash solution. Additional ACD Multiplex HRP blocker and wash steps were performed to ensure that all HRP activity was quenched. Slides were open washed 3 times with 1X Bond wash buffer, and incubated with Donkey anti-Rat-HRP (1:1000) (Jackson Immuno-Research, 712-035-153). CD69 ICC was detected by incubation with PerkinElmer Opal-690 (1:1500) in 1X amplification buffer for 30 minutes at RT. Excess dye was rinsed off by washing 8 times with 1X Bond wash solution. Slides were counterstained with spectral DAPI for 5 minutes at RT. Excess DAPI was rinsed off by 5 washes with DI water. Finally, the slides were cover slipped with Prolong Gold anti-fade reagent (Life Technology Cat # P36930). Antibodies used in this study are listed below (Table 2).

**Table 2:** Antibodies for ICC used in this study.

Antibody Name	Reference Number	Opal Fluor	Reference Number
Rat anti-murine CD69	R&D Systems (Clone#310116) Cat # MAB23861	Opal-520	PerkinElmer FP1487001KT
Rabbit anti-murine Notch1	Cell Signaling Technologies (Clone-D1E11) Cat # 3608	Opal-690	PerkinElmer FP1497001KT
mono-Rat IgG1, K	BD Pharmingen (clone R3-34) Cat # 553922	Opal-520	PerkinElmer FP1487001KT
mono-Rabbit IgG1	Cell Signaling Technologies (Clone-DA1E) Cat # 3900S	Opal-690	PerkinElmer FP1497001KT
Rat anti-human CD209	LifeSpan BioSciences, Inc (Clone: h209) Cat # LS-B3782/73607	Opa-520	PerkinElmer FP1487001KT
mono-Rat IgG2a, k	Pharmingen (Clone R35-95) Cat # 553927	Opal-520	PerkinElmer FP1487001KT

### Hematoxylin and eosin (H & E) staining

H & E staining was performed by incubation with 3X 5 minutes each in xylene, followed by re-hydration with two 1 minute each with 100%, 95% and 1X 70% Ethanol (EtOH) and then washed with running tap water for 1 minute. Slides were stained with Hematoxylin (American Master Tech, Gill’s III, HXGHE3GAL) for 8 minutes and washed with running tap water for 1 minute. After staining, slides were washed in acid alcohol (0.5% HCl in 95% EtOH) and washed again in running tap water for 1 minute. Bluing reagent (Richard Allan Scientific, 7301) was added for 1 minute and excess washed with running tap water for 1 minute. Slides were stained with Eosin (American Master Tech, STE0157) (dilute 1:1 with 95% EtOH) for 30 seconds and dehydrated 2 times for 1 minute each in 95%, 100% EtOH and Xylene.

### Image acquisition, processing and quantification

Brightfield images were acquired with a Nanozoomer 2.0 HT using NDP scan 3.2.12 software (Hamamatsu).

Confocal images were acquired using a Leica TSC SP8 fitted with a 100x oil-immersion objective lens (HC PlanApochromat 100x/1.40 oil STED white, NA = 1.40). Confocal images were obtained in sequential acquisition mode using HyD detectors. The pinhole was set at one Airy unit. 3D scanning was performed with 8 frames averaging and bidirectional scanning using excitation/emission wavelengths accordingly to Table 3:

**Table 3:** Fluorophores and wavelengths used for target detection.

Fluorophore	Excitation wavelength (nm)	Emission wavelength
DAPI	405 (UV)	410–441
OPAL-520	488 (Argon)/494	491-528/499-535
OPAL-570	550	559–587
OPAL-620	588	595–647
OPAL-690	670	674–727

For the multiplex imaging, 3 sequential scans were created to avoid overlapping spectra: sequence 1–405 nm (UV) together with 670 nm excitation, sequence 2–494 nm together with 588 nm excitation, sequence 3–550 nm excitation.

Projected images were generated by collecting maximum pixel intensity of the entire z-stack frames into a single frame. All quantified images were acquired and processed simultaneously by using identical confocal settings. All post acquisition processing and analysis was performed with ImageJ (NIH). For analysis, z-stacks of unprocessed images were used. For puncta count, a cytoplasmic mask was created and the number of puncta was extracted with automatic “analyze particles” algorithm. To determine MFI measurements, ImageJ was used to select 2–3 random regions of interest (ROI) within each cell present in the image. The ROI values for each cell were averaged, and then the background signal level was subtracted from these.

Each point on the graphs represents the mean value (either number of puncta or MFI) for one cell. Signal from each cell in the image was counted in order to do not induce bias.

### Flow cytometry analysis

At harvesting, 1x10^5^ cells were placed in V-bottom 96 well plates, spun at 350 RCF for 5 minutes at 4ºC, and the supernatant was discarded. Cell pellets were washed with 1X HBSS (Gibco cat # 14175–095) complemented with 2% hiFBS (hyClone cat # SH3008803HI). Cells were spun as previously described and the supernatant removed. Next, the plate was placed on ice, pellets were resuspended in FC-blocking solution composed of FACS buffer (HBSS, 2% hiFBS, 0.05% sodium azide, 0.5% Bovine Serum Albumin) and 1:100 dilution of mouse FC blocking purified Rat anti-mouse CD16/CD32 (BD Pharmingen cat # 553142) and then incubated on ice for 10 minutes. Cells were then stained for 20 minutes on ice, by addition of staining solution composed of FACS buffer and the antibodies as reported in the table below (Table 4).

**Table 4:** Antibodies for FACS analysis used in this study.

Antibody	Reference	Dilution
APC/Cy7 anti-mouse CD25	BioLegend cat # 102026	1:100
PE anti-mouse Notch1	BD Pharmingen cat # 553989	1:100
APC anti-mouse CD69	eBioscience cat # 17-0691-82	1:100
eFluor450 anti-mouse CD62L	eBioscience cat # 48-0621-82	1:100
FITC anti-mouse CD8a	BD Pharmingen cat # 553031	1:200

Cells were spun as before and supernatant discarded. 3 washes with FACS buffer were then performed. Finally, cells were resuspended in 100 uL of FACS buffer to which fresh 7-AAD (BD Pharmingen 51-68981E) was added.

Flow cytometry was performed on LSR II machine (BD) and analysis done with FlowJo software.

### RNA extraction and RT-qPCR

2x10^6^ cells per sample were used for RNA extraction with Qiagen RNeasy mini kit (cat # 74104) accordingly to manufacturer instructions. 500 ug of isolated RNA was subjected to reverse transcription with High Capacity cDNA Reverse Transcription kit (AB cat # 4368814).

The obtained cDNA was pre-amplified using Taqman PreAmp Master Mix (AB cat # 4391128). Finally, qPCR was performed with Taqman Gene Expression Master Mix (cat # 4369016) on Viia7 machine (AB). Taqman assays used to detect the targets of interest were purchased from ThermoFisher Scientific and are reported in the table below (Table 5).

**Table 5:** Taqman probes used in this study.

Taqman assay	Reference
CD69	Mm01183378_m1
Rplp0	Mm00725448_s1
Rlp19	Mm02601633_g1
ActB	Mm00607939_s1
Notch1	Mm00435249_m1
Notch1	Mm00627185_m1
Notch1	Mm00627192_m1
Notch1	Mm03053614_s1
Notch1	Mm00435245_m1

The delta-delta Ct method was used to evaluate gene expression. Cts were normalized against the geometric mean of the housekeeping genes *Rplp0*, *RLP19* and *ActB*. Due to its low level of expression, *Notch1* mRNA was measured with multiple probes to ensure accurate measurement. The geometric mean of the Cts for Notch1 probes (min 2, max 5) was used for subsequent gene expression calculation.

## Results

### Cytospin slide preparation allows usage of low cell numbers and maintains cell morphology

After isolation from mouse spleen and lymph nodes, purified CD8+ T cells were activated *in vitro* via plate-bound anti-CD3e antibody. Purity, viability and differentiation state of isolated cells were checked by flow cytometry as described in Materials and Methods (Flow cytometry analysis chapter), by accessing 7-AAD staining and surface expression of CD8, CD62L, CD69 and CD25. A representative FACS analysis, including gating strategy, is shown in S1 Fig.

Our first goal was to select a cell preparation method that would allow the use of a small number of cells, with minimum loss, that would maintain tissue morphology for pathology evaluation, that would be suitable for subsequent ISH and ICC procedures, and require a minimum handling time. To this aim we selected cytospin cell preparation.

**Fig 1:**
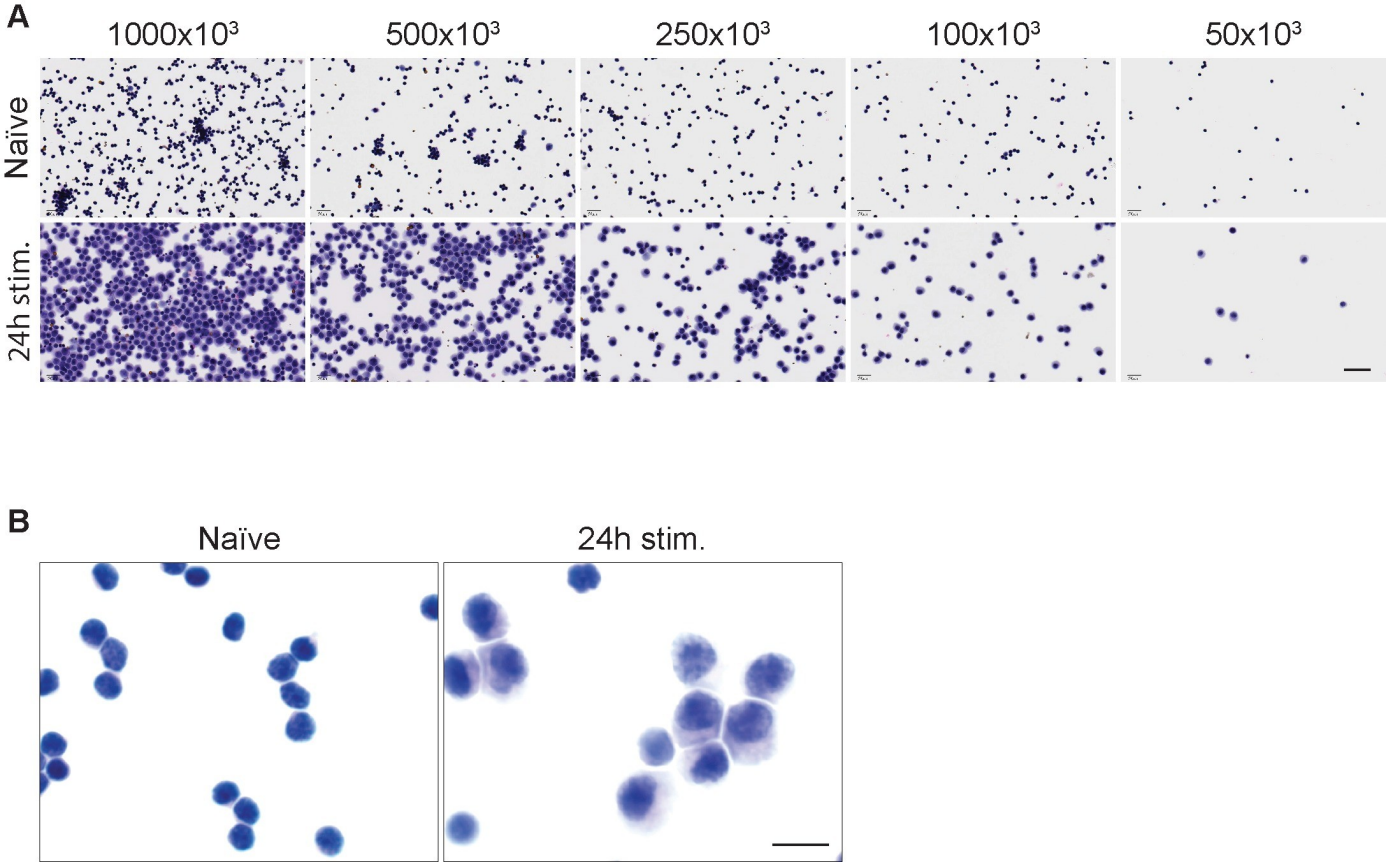
Cytospin CD8+ T cells preparation. (A) Determination of optimal cell density for cytospin slides preparation with both naïve lymphocytes (first row) and lymphocytes stimulated for 24 hours with plate-bound anti-CD3e (second row). Images were taken with the same magnification. Scale bars = 40 μm. (B) Hematoxylin and Eosin staining of cytospin cell preparations (at 2.5x10^5^ cells/slide) showing that cells maintain natural morphology. In the left panel murine naïve CD8+ T cells, in the right panel CD8+ T cells after 24 hours of in vitro stimulation are shown. Images were taken with the same magnification. Scale bars = 10 μm.

Non-stimulated (naïve) and stimulated cells for 24 hours were harvested and prepared for cytospin as described in Materials and Methods (cytospin chapter), and subsequently stained with hematoxylin and eosin (H&E staining, Fig 1). Serial dilutions were used to determine the optimal cell density to obtain a monolayer of evenly distributed cells (Fig 1A). Exceedingly high cell density led to the formation of cell agglomerates, while a too diluted cell preparation resulted in an uneven cell distribution. Particular attention in the determination of the optimal cell density should be used when working with different cell types, or samples with different morphologies. As shown in Fig 1A, we determined that a cytospin preparation of 2.5x10^5^ cells/slide, in a volume of 250 uL (i.e. a density of 1x10^6^/mL) would give the best distribution for both naïve cells and for cells subjected to 24 hours of stimulation. However, we found that acceptable results could be obtained with as few as 5x10^4^ cells/slide.

Cytospun cells maintained normal morphology, that was visibly different between naïve cells and cells subjected to 24 hours of stimulation (Fig 1B).

### Development of dual ISH/ICC protocol with RNAscope on cytospin samples

In Materials and Methods, we provide protocols for both chromogenic single ISH and dual ISH/ICC and fluorescent single ISH and dual ISH/ICC. We also provide guidelines for performing manual and fully automated procedures. The results shown here were produced with our fluorescent dual ISH/ICC method on a fully automated platform (Leica Bond RX).

#### Sample RNA quality control

**Fig 2:**
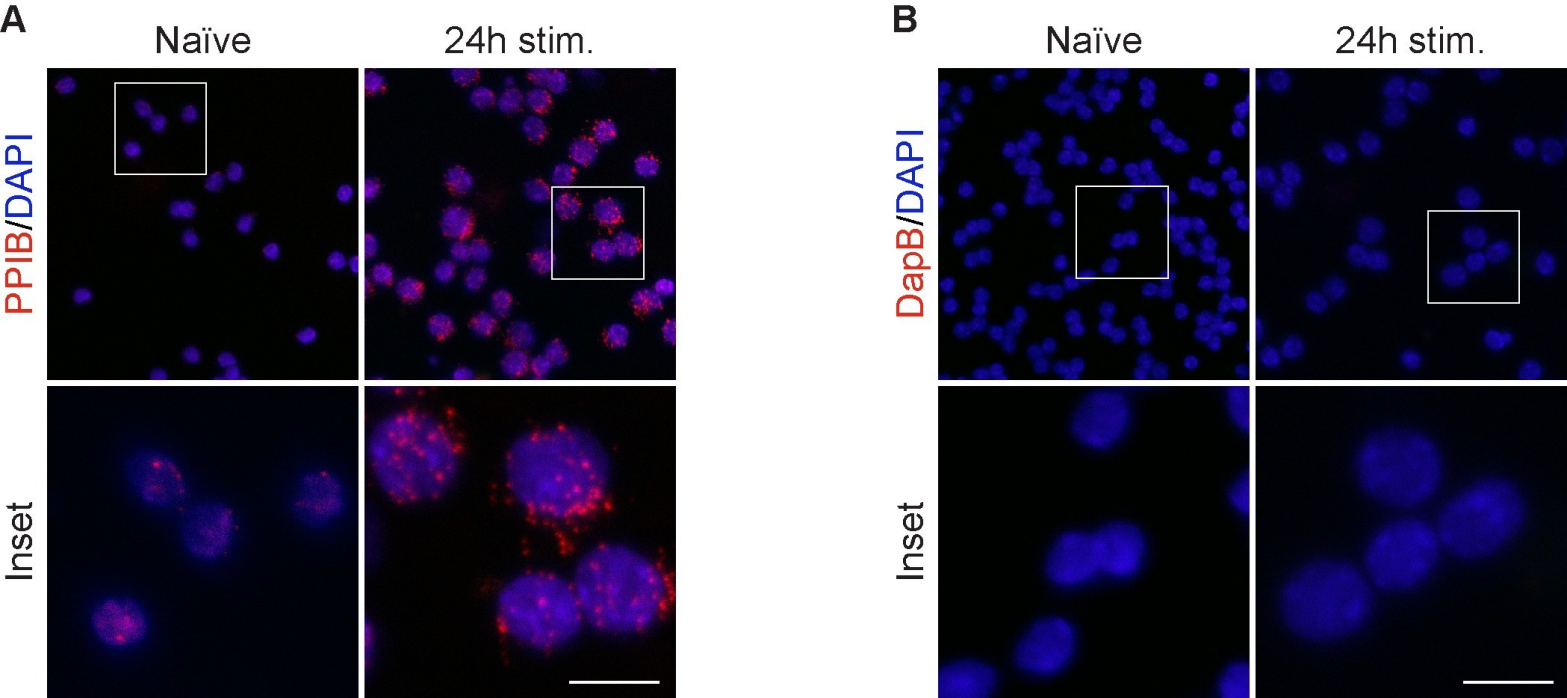
RNA quality control for ISH. (A) RNAscope probe targeting Peptidil-prolyl cis-trans isomerase B (PPIB) RNA on naïve and stimulated CD8+ T cells is used to access RNA quality in cytospin samples. (B) Negative control for RNAscope PPIB staining using a non-targeted probe for DapB (dihydrodipicolinate reductase, from B.subtilis). Scale bar is equal to 10 μm.

Preservation of RNA integrity within the samples is essential for ISH performance and deterioration can occur during extended manipulation for sample preparation. For this reason, RNA integrity was verified by performing ISH for the ubiquitously expressed peptidylprolyl isomerase B (*Ppib*) mRNA on cytospin samples (Fig 2A) using a single fluorescent ISH procedure, described in Materials and Methods (Automated procedures- Fluorescent single ISH procedure). As negative control, we selected an off-target ISH probe that recognizes B.subtilis dihydrodipicolinate reductase (*dapB*) mRNA (Fig 2B). While a distinct red punctate pattern is visible for *Ppib* staining, no signal is observed with the *dapB* control. This data supports the use of cytospin as a quick and safe suspension cell preparation.

#### Dual ISH/ICC

**Fig 3:**
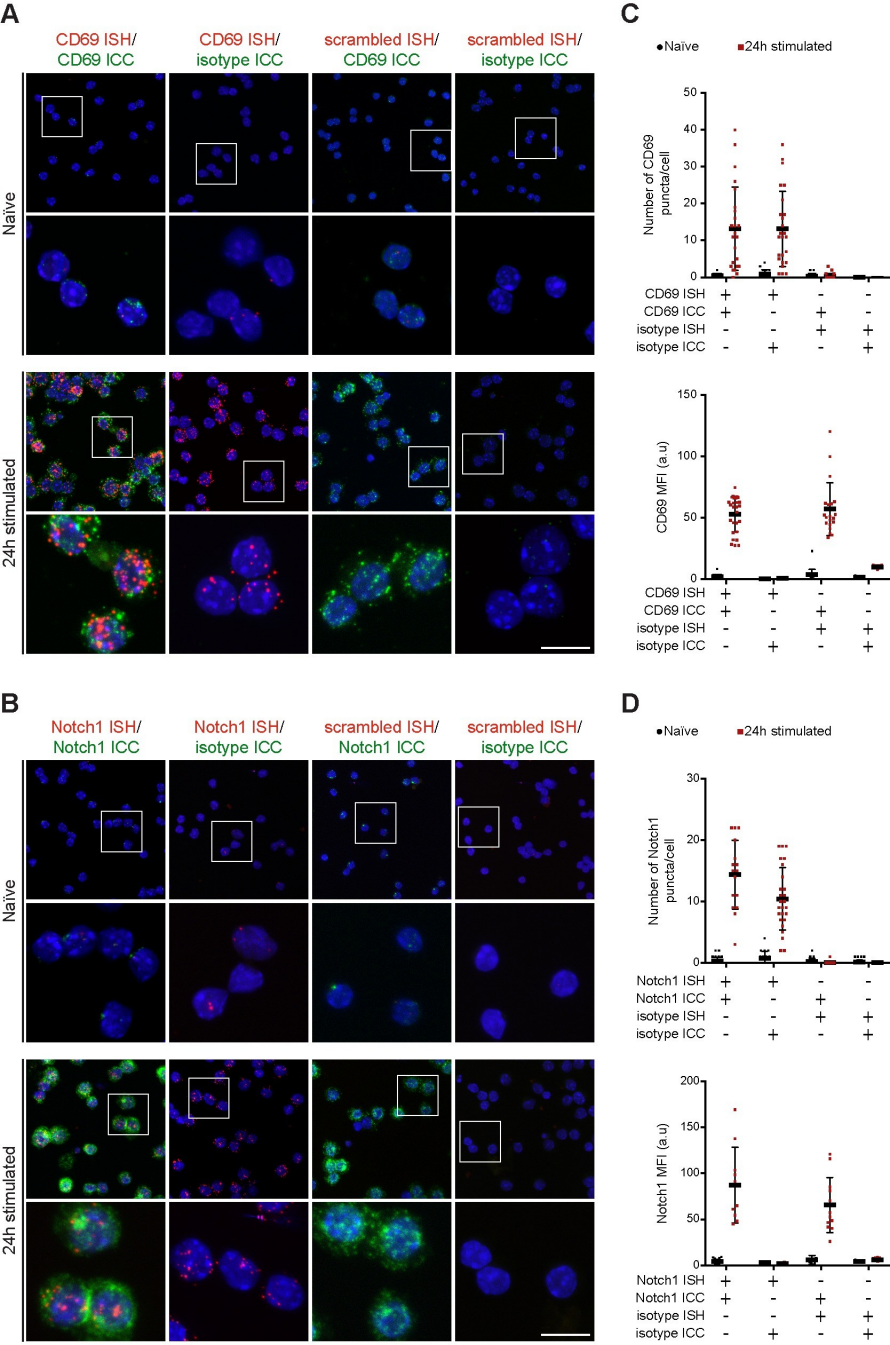
Development of dual ISH/ICC protocol for CD69 and Notch1. (A) Dual ISH/ICC for CD69. CD69 RNAscope probe was used to target CD69 RNA (red signal), while rat anti-CD69 antibody was used to detect expression of CD69 protein (green signal). First column shows dual positive staining for CD69 ISH/ICC, second column shows ISH-only control in which anti-CD69 probe was used in combination with a rat isotype for ICC. Images in the third column shows an ICC-only control (scrambled ISH probe combined with rat anti-CD69 antibody). The fourth column shows double negative control (scrambled sequence probe and isotype). Scale bar is equal to 10 μm. (B) Dual ISH/ICC for Notch1. In columns from the left: dual ISH/ICC, ISH-only control, ICC-only control, dual negative control. Scale bar is equal to 10 μm. (C) Signal quantification for dual CD69 ISH/ICC. RNA signal is quantified as number of dots per cell. Protein expression is quantified as signal intensity per cell (median fluorescence intensity, MFI). (D) Signal quantification for dual Notch1 ISH/ICC. RNA signal is quantified as number of dots per cell. Protein expression is quantified as signal intensity per cell (median fluorescence intensity, MFI). Each symbol on the graph represents the mean value of one cell, data are collected from images of multiple experiments and error bars represent SEM.

Performance of dual ISH/ICC protocol (see Materials and Methods - Automated procedures—Fluorescent dual ISH/ICC procedure) was evaluated for two targets known to be differentially regulated in naïve and stimulated CD8+ T cells: CD69 and Notch1. Both CD69 and Notch1 are induced upon T cells activation, while being absent or very low in naïve cells [19, 23, 24]. Expression of each target was evaluated in separate sets of dual ISH/ICC experiments presented in Fig 3: panel A shows CD69 dual ISH/ICC, panel B shows Notch1 dual ISH/ICC. ISH was performed with probes against *Cd69* or *Notch1*, ICC was conducted with Rat anti-murine CD69 or Rabbit anti-murine NOTCH1. As negative controls, ISH/ICC was performed with scrambled-sequence probe and species-specific antibody isotype. Additional controls were introduced by performing specific-probe ISH combined with isotype control ICC and vice-versa. These series of controls are informative on possible crosstalk or non-specific signal arising during ISH/ICC combination.

mRNA detection is marked by a distinct punctate red staining, visible for both probes in stimulated cells, while signal in naïve cells is minimal. A green signal marks expression of target proteins and correlates with the presence of mRNA, as expected (Fig 3, panels A and B, first columns). Substitution of primary antibodies with the respective species-specific isotypes did not generate any appreciable signal, thus confirming the specificity of the antibodies selected and the fidelity of the protocol applied (Fig 3, panels A and B, second columns). Scrambled sequence probes were used as negative controls to verify that signal arising from stimulated cells was indeed dependent on specific probe hybridization to the target mRNA. ISH negative controls did not show any red signal (Fig 3, panels A and B, third columns). Importantly, we detected no evident crosstalk between ISH and ICC procedures. Finally, no non-specific signal was detected in slides where scrambled-sequence probes and isotypes were used (Fig 3, panels A and B, fourth columns).

To prove the robustness of the assay developed and its direct applicability to different cytological specimens, human monocyte-derived dendritic cells were subjected to dual ISH/ICC for detection of CD11c mRNA and CD209 protein, two well known phenotypic markers of this cell population [25]. Similarly to CD8+ T cells, the assay proved to be sensitive and specific (S2 Fig). Importantly, the procedure to perform dual ISH/ICC on monocyte-derived dendritic cells did not require further optimization compared to murine CD8+ T cells.

#### Quantification

Dual ISH/ICC is inherently a semi-quantitative technique. RNAscope technology produces single puncta that correspond to individual nucleic acid molecules. Additionally, MFI quantification can be applied to evaluation of protein expression from the ICC signal.

As a proof of concept, we quantified the number of puncta and the MFI of ICC signals from dual ISH/ICC and respective controls for both CD69 (Fig 3C) and Notch1 (Fig 3D). For both targets we observed an increased ISH labeling as quantified by the number of puncta in cells after 24 hours in stimulation compared to naïve cells, but no signal in negative controls. Similarly, we detected a clear increment in ICC signal (MFI) in cells upon stimulation for 24 hours compared to baseline, and no non-specific staining in negative controls.

### Sensitivity of dual ISH/ICC allows the detection of subtle differences between samples

The dual ISH/ICC protocol shown here has demonstrated to be robust and reliable in the detection of two well expressed markers during lymphocytes activation. We next tested if this method is sensitive enough to discriminate more subtle differences in expression patterns. To this aim, we investigated the expression of CD69 and Notch1 during the first hours of lymphocyte stimulation and attempted to discriminate the kinetics of mRNA and protein expression of these two targets. Primary CD8+ T cells were isolated from mouse spleen and lymph nodes. Purified lymphocytes were stimulated *in vitro* via plate-bound anti-CD3e for 1, 2, 3 and 4 hours. Samples were cytospun and dual ISH/ICC performed as previously described. For simplicity, only dual positive images are shown here while the previously described experimental controls (ISH/isotype, scrambled-ISH/ICC, and scrambled-ISH/isotype) are reported in S3 Fig.

**Fig 4:**
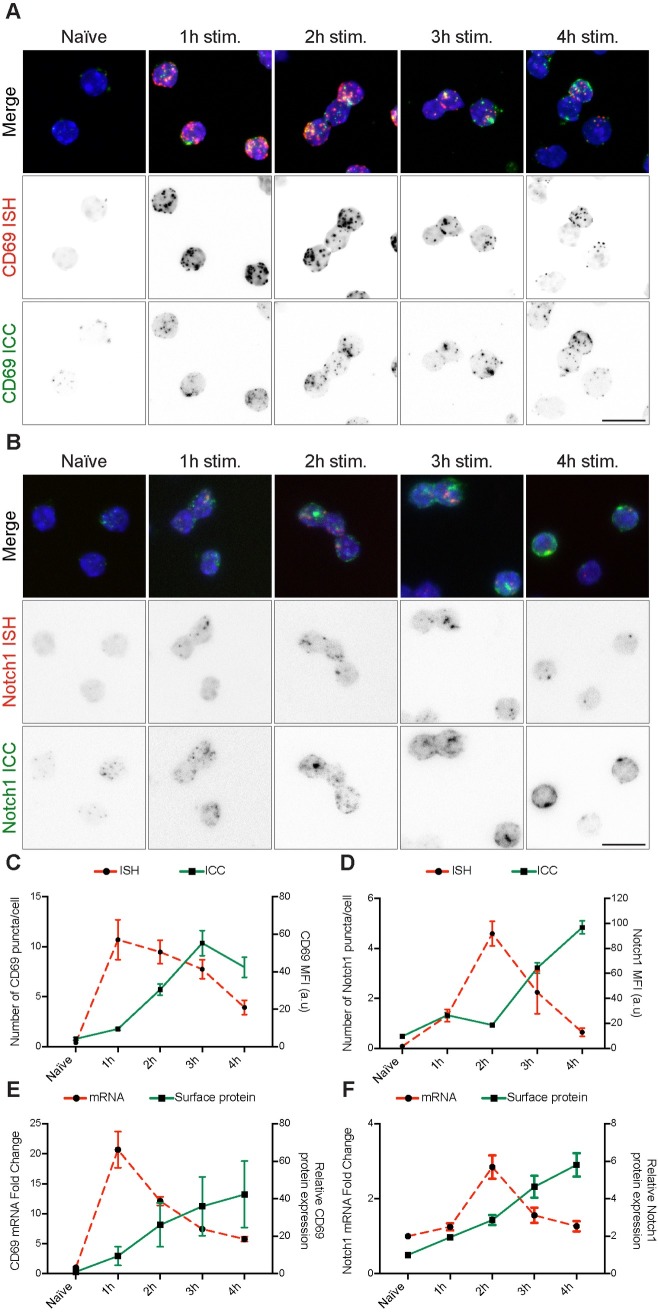
CD69 and Notch1 expression kinetics during CD8+ T cell activation. Dual CD69 ISH/ICC (A) and dual Notch1 ISH/ICC (B) on naïve and activated lymphocytes via plate-bound anti-CD3e antibody for 1 to 4 hours. RNAscope probe signal shown in red, protein signal shown in green. Channels are split in each panel to help visualization of the expression kinetics of the targets (First lane: merged signal, second lane: RNAscope signal, third lane: protein signal). Scale bar is equal to 10 μm. (C)-(D) ISH/ICC signal quantification from panels A and B respectively. RNA signal is quantified as number of dots per cell. Protein expression is quantified as signal intensity per cell (median fluorescence intensity, MFI). Mean values are plotted, error bars represent SEM. (E)-(F) Comparison of results obtained with dual ISH/ICC protocol against standard quantification methods for RNA and protein expression for CD69 and Notch1 respectively. RNA expression is measured by RT-qPCR with Taqman assays and flow cytometry analysis is conducted for surface markers within unfixed living cells (median fluorescence intensity, MFI). Mean values of 4 replicate experiments for RNA and 7 replicates for flow cytometry are plotted, data are represented as normalized on naive cells values for each experiment, for both RNA and protein expression. Error bars represent SEM.

CD69 is one of the earliest activation markers expressed by lymphocytes [19]. Not surprisingly, dual ISH/ICC for CD69 shows abundant mRNA expression already after 1 hour of *in vitro* stimulation, as compared to naïve cells. Interestingly, mRNA abundance seems to slowly decline over time. CD69 protein accumulation on the cell surface starts as early as 1 hour post-stimulation and continues throughout the time course (Fig 4A). In contrast, Notch1 exhibits slower activation and expression. *Notch1* mRNA production seems to peak after 2 hours of stimulation *in vitro* and rapidly declines over time, while accumulation of protein on the cell surface becomes evident from the third hour of stimulation (Fig 4B). Quantification of the signals supports the qualitative impression of these images (Fig 4C and 4D, cell population images are shown in S4 Fig).

Performance of dual ISH/ICC was then compared with commonly used methods to determine mRNA and surface protein expression.

To this aim, naïve and stimulated CD8+ T cells for 1 to 4 hours were analyzed by flow cytometry and RT-qPCR (taqman assay). By these methods *Cd69* mRNA was highly expressed after 1 hour of *in vitro* stimulation and slowly decreased over time, while protein on the surface accumulated progressively (Fig 4E). *Notch1* mRNA was expressed within 2 hours of stimulation, while protein accumulated significantly on the cell surface within 3 hours (Fig 4F). Measurement of mRNA and protein signals from ISH/ICC correlates with RT-qPCR and FACS counts respectively, confirming that the dual ISH/ICC protocol can reliably measure mRNA and protein levels *in situ*.

### Multiplex ISH/ICC

Oftentimes multiple targets need to be visualized within the same cell. The robustness of the assay here developed allows multiplexing as far as separate fluorescent channels are available for probes and antibodies combinations.

As proof of concept a 5-plex experiment was performed (as described in Materials and Methods—Automated procedures—Fluorescent multiplex ISH/ICC procedures), in which two ISH, two ICC and DAPI were combined on the same sample. Specifically, Notch1 ISH/ICC was combined with CD69 ISH/ICC on naïve or 24 hours stimulated CD8+ T cells. This combination allows the detection of Notch1 in relation to activated CD8+ T cells (CD69+) by both mRNA and protein levels. All probes and antibodies were tested individually before multiplex was performed.

**Fig 5:**
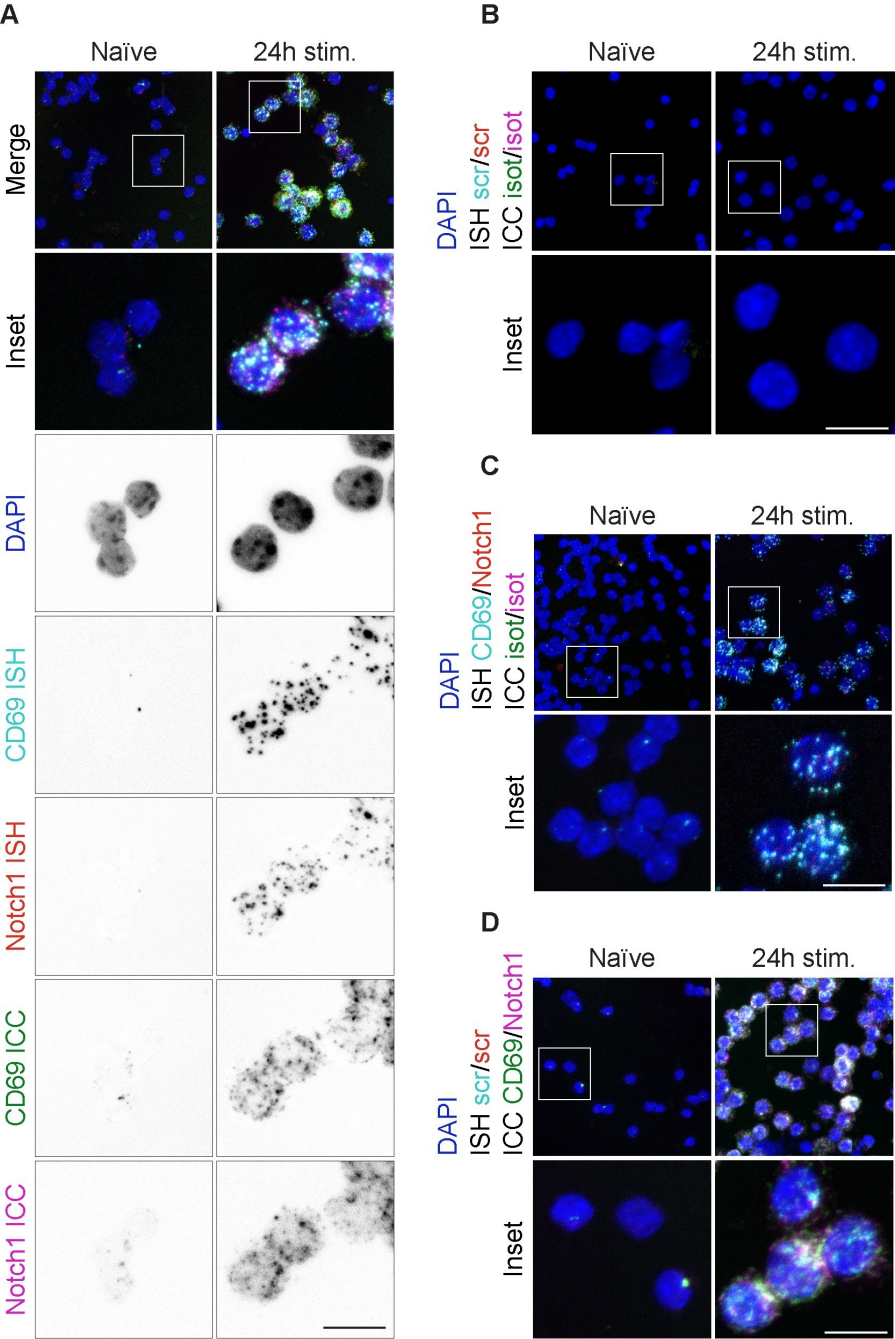
Multiplex ISH/ICC development. Multiplex ISH/ICC assay for detection of 4 targets simultaneously on naïve and stimulated lymphocytes for 24 hours. (A) Detection of CD69 and Notch1 proteins and mRNAs. Channels are split below merged image to help visualization. (B) Multiplex negative control. Scrambled-sequence probes and isotype antibodies were used. (C) ISH-only control in which CD69 and Notch1 targeted probe were used in combination with species-specific antibody isotype controls for both target ICC. (D) ICC-only control in which scrambled-sequence probes were used in combination with specific anti-CD69 and anti-Notch1 antibodies for ICC. White color in the images corresponds to actual overlapping signals from different probes and/or antibodies: in fact to avoid bleed-through of fluorescence emission, we combined sequential scanning to ensure no signal bleed-through between channels. Scale bar is equal to 10 μm.

As previously observed, naïve cells express very little CD69 and Notch1, while activated cells robustly sustain mRNA and protein expression of both targets (Fig 5A). As control, multiplex ISH/ICC was performed substituting targeted probes with scrambled-sequence ones and specific antibodies with isotypes (Fig 5B). The specificity of the ICC signals was tested by replacing CD69 and Notch1 antibodies with respective isotypes for the ICC part of the multiplex protocol, thus revealing uniquely the mRNA signals (Fig 5C). Finally, substitution of specific probes with scrambled-sequence ones for both CD69 and Notch1 in the ISH portion of the protocol reveals uniquely ICC derived signal (Fig 5D).

Also in this case, signals from each target can be quantified, as shown in S5 Fig.

## Discussion

This work provides guidelines to perform quantitative ultrasensitive RNAscope ISH combined with ICC on cytospin cell preparations. Methods exist for combining ISH and IHC on FFPE tissue sections or non-adherent cell types can be processed into pellets and treated as tissue sections as well. Alternatively, procedures can be performed in solution, and cells subsequently immobilized on a support (glass-slide or glass-bottom plate). However, those processes require a high number of cells and extensive handling, which preclude their use for cytological specimens with small cell numbers. In order to overcome these obstacles, we sought to develop a method to perform combined ISH/ICC on paucicellular non-adherent specimens.

Cytospin is an excellent tool for the preparation of paucicellular samples for downstream protein or nucleic acid analysis because (i) the procedure is rapid, thus limiting the risk of target degradation, (ii) morphology of the cells is maintained, permitting precise pathology evaluation, (iii) optimization of cell concentration required for adequate cell density on the slide is simple. Importantly, cytospin is advantageous also for non-adherent cell types that can generate highly cellular specimens as it allows a significant reduction of the material needed, which may help reduce animal numbers or allow for additional endpoints using the same amount of starting material. Paraffin embedded tissue samples and cytospin preparations differ in several ways. Accordingly, protocols for *in situ* detection of RNA or protein in cytospin preparations require specific adaptations compared to the standard FFPE protocols from which they derive. Notably, cytospin samples are much more fragile than typical tissue sections. In addition, FFPE tissue sections, due to the extensive processing during tissue embedding and due to the sectioning process itself do not usually require specific steps for permeabilization of cell or compartment membranes, whereas cell preparations may require them for visualization of intracellular targets. In this manuscript, we provide details of such adaptations and also describe how certain pre-programmed steps in existing immunostainer software that are designed for staining FFPE tissue sections can be repurposed to accommodate cytospin preparations.

Data presented demonstrate that the ISH/ICC protocols outlined in this study are readily adaptable to a variety of targets and cell types without the need of extensive customization. Furthermore, the techniques described are highly sensitive and quantifiable, allowing the reliable and quantitative detection of subtle differences in expression kinetics of the studied targets. RNAscope produces single puncta that correspond to individual nucleic acid molecules [6]. Determination of the number of puncta per cell gives a direct measurement of mRNA expression. Similarly, MFI calculation for the ICC signals corresponds to the relative protein expression. In practicality, adjustments of the ISH signal quantification methodology may be required for highly expressed genes because ISH puncta can overlap and fuse resulting in inaccurate quantifications. In such cases, MFI measurement of the ISH signal may also be used for relative measurement of mRNA expression. Notably, quantification of ISH/ICC compares well with semi-quantitative methods commonly used, such as Taqman RT-qPCR and flow cytometry. ISH/ICC has several advantages over semi-quantitative methods like RT-qPCR or generic flow cytometry, including: (i) the possibility to evaluate cell morphology, (ii) both mRNA and protein expression can be measured within the same cell population in a single experiment, (iii) information about subcellular localization can also be collected, (iv) the analysis of single cells (as opposed to bulk populations) enables the identification and evaluation of expression patterns within cell populations, *in situ*. In addition, qPCR measurement of gene expression between very different cell populations may require extensive validation of appropriate housekeeping targets used for data normalization [2], while ISH/ICC does not.

The understanding of the molecular footprints of pathologies requires the analysis of multiple biomarkers within the same specimen. Technologies that allow multiplexed detection of several targets in the same sample are therefore needed. We show the potential for multiplexing of our protocol by performing a 5-plex experiment (2 mRNA, 2 protein targets and DAPI for visualization of cell nuclei). When multiplexing using protocols such as outlined here, it is important to test each probe or antibody separately and compare the observations with those of the multiplexed protocol. Likewise, fluorescent channel assignment must be chosen strategically. These two measures are necessary because staining is performed in several sequences that may interfere.

When comparing manual with automated procedures, we did not observe any qualitative differences. This indicates that sophisticated instrumentation is not required for the type of experiment described, enabling many laboratories to implement these techniques. At the same time, the procedures are scalable for larger facilities that have to process higher volumes of slides or a need to run similar studies repeatedly. The provided automated protocols increase throughput and reproducibility as well as speed. In fact, the automated protocol here described allows processing of samples from tissue harvesting to imaging in as few as 2 days.

In conclusion, we developed a method to perform quantitative ultrasensitive multiplex ISH/ICC on cytospin samples overcoming limitations of non-adherent cytological samples that may otherwise prevent their use in this type of experiment. To allow easy customization depending on the specific needs of a particular project or laboratory, we provide detailed guidelines for performing such assays in brightfield chromogenic or darkfield fluorescent formats, as well as in manual settings or on automated staining platforms.

## Supporting information

S1 Checklist NC3Rs ARRIVE guidelines checklist. (PDF)

S1 Fig FACS analysis of purified naïve and stimulated CD8+ Tcells. Representative FACS analysis of purified lymphocytes. (A) Lymphocytes were first identified in forward (FSC-A) and side scatter (SSC-A) dot plot to exclude debris, percentage represents gated fraction of total cells. (B) Of the previous population, only living (7-AAD negative) CD8+ T cells (CD8 positive) were considered for marker analysis. Please note that about 97% of purified living cells were CD8+. (C) The previous CD8+ population was analyzed for expression of CD69, CD25 and CD62L to access stimulation efficacy.
(TIF)

S2 Fig Dual CD11c ISH/ CD209 ICC on human monocyte-derived dendritic cells. Representative images for dual ISH/ICC on human monocyte-derived dendritic cells. First row shows populations, second row is the zoomed in inset shown in the population images (white square). For inset images, split channels for each detector are shown in grey tone images to help visualization (third, fourth and fifth row).
(TIF)

S3 Fig Time course controls. While only dual positive images are shown in Fig 4 for simplicity, here are shown all the controls performed. (A) Detection of CD69. (B) Detection of Notch1. In both panels: first row shows dual positive staining for ISH/ICC, second row shows ISH-only control in which specific probe was used in combination with an isotype for ICC. Images in the third row shows an ICC-only control, i.e. scrambled ISH probe combined with isotype antibody. The fourth row shows double negative control (scrambled sequence probe and isotype).
(TIF)

S4 Fig Cell populations for time course. Cell populations from which insets shown in Fig 4 were taken. Top row shown dual ISH/ICC for CD69, bottom row shows dual ISH/ICC for Notch1.
(TIF)

S5 Fig 5-plex experiment quantification. Quantification of signals for ISH and ICC for multiplex experiment.
(TIF)

## References

[1] Goossens N, Nakagawa S, Sun X, Hoshida Y. Cancer biomarker discovery and validation. Transl Cancer Res. 2015;4(3):256–69. 10.3978/j.issn.2218-676X.2015.06.04 ; PubMed Central PMCID: PMCPMC4511498. 26213686 10.3978/j.issn.2218-676X.2015.06.04 PMC4511498

[2] Guenin S, Mauriat M, Pelloux J, Van Wuytswinkel O, Bellini C, Gutierrez L. Normalization of qRT-PCR data: the necessity of adopting a systematic, experimental conditions-specific, validation of references. J Exp Bot. 2009;60(2):487–93. 10.1093/jxb/ern305 . 19264760 10.1093/jxb/ern305

[3] Cassidy A, Jones J. Developments in in situ hybridisation. Methods. 2014;70(1):39–45. 10.1016/j.ymeth.2014.04.006 . 24747923 10.1016/j.ymeth.2014.04.006

[4] Qian X, Lloyd RV. Recent developments in signal amplification methods for in situ hybridization. Diagn Mol Pathol. 2003;12(1):1–13. . 12605030 10.1097/00019606-200303000-00001

[5] Itzkovitz S, van Oudenaarden A. Validating transcripts with probes and imaging technology. Nat Methods. 2011;8(4 Suppl):S12–9. 10.1038/nmeth.1573 ; PubMed Central PMCID: PMCPMC3158979. 21451512 10.1038/nmeth.1573 PMC3158979

[6] Wang F, Flanagan J, Su N, Wang LC, Bui S, Nielson A, et al RNAscope: a novel in situ RNA analysis platform for formalin-fixed, paraffin-embedded tissues. J Mol Diagn. 2012;14(1):22–9. 10.1016/j.jmoldx.2011.08.002 ; PubMed Central PMCID: PMCPMC3338343. 22166544 10.1016/j.jmoldx.2011.08.002 PMC3338343

[7] Wang H, Wang MX, Su N, Wang LC, Wu X, Bui S, et al RNAscope for in situ detection of transcriptionally active human papillomavirus in head and neck squamous cell carcinoma. J Vis Exp. 2014;(85). 10.3791/51426 ; PubMed Central PMCID: PMCPMC4145807. 24637627 10.3791/51426 PMC4145807

[8] Alexander Franks EA, Slavov Nikolai. Post-transcriptional regulation across human tissues. PLOS Computational Biology. 2017 10.1371/journal.pcbi.1005535 28481885 10.1371/journal.pcbi.1005535 PMC5440056

[9] Duraiyan J, Govindarajan R, Kaliyappan K, Palanisamy M. Applications of immunohistochemistry. J Pharm Bioallied Sci. 2012;4(Suppl 2):S307–9. 10.4103/0975-7406.100281 ; PubMed Central PMCID: PMCPMC3467869. 23066277 10.4103/0975-7406.100281 PMC3467869

[10] Matos LL1 TD, de Matos MG, da Silva Pinhal MA. Immunohistochemistry as an Important tool in biomarkers detection and clinical practice. Biomark Insights. 2010;5:9–20. Epub 2010 Feb 9. PubMed Central PMCID: PMCPMC2832341. 20212918 10.4137/bmi.s2185 PMC2832341

[11] Stack EC, Wang C, Roman KA, Hoyt CC. Multiplexed immunohistochemistry, imaging, and quantitation: a review, with an assessment of Tyramide signal amplification, multispectral imaging and multiplex analysis. Methods. 2014;70(1):46–58. 10.1016/j.ymeth.2014.08.016 . 25242720 10.1016/j.ymeth.2014.08.016

[12] Blom S, Paavolainen L, Bychkov D, Turkki R, Maki-Teeri P, Hemmes A, et al Systems pathology by multiplexed immunohistochemistry and whole-slide digital image analysis. Sci Rep. 2017;7(1):15580 10.1038/s41598-017-15798-4 ; PubMed Central PMCID: PMCPMC5686230. 29138507 10.1038/s41598-017-15798-4 PMC5686230

[13] Grabinski TM, Kneynsberg A, Manfredsson FP, Kanaan NM. A method for combining RNAscope in situ hybridization with immunohistochemistry in thick free-floating brain sections and primary neuronal cultures. PLoS One. 2015;10(3):e0120120 10.1371/journal.pone.0120120 ; PubMed Central PMCID: PMCPMC4368734. 25794171 10.1371/journal.pone.0120120 PMC4368734

[14] Anderson CM, Zhang B, Miller M, Butko E, Wu X, Laver T, et al Fully Automated RNAscope In Situ Hybridization Assays for Formalin-Fixed Paraffin-Embedded Cells and Tissues. J Cell Biochem. 2016;117(10):2201–8. 10.1002/jcb.25606 ; PubMed Central PMCID: PMCPMC5132049. 27191821 10.1002/jcb.25606 PMC5132049

[15] Qamar I1 RS, Mehdi G1, Maheshwari V1, Ansari HA1, Chauhan S1. Utility of Cytospin and Cell block Technology in Evaluation of Body Fluids and Urine Samples: A Comparative Study. J Cytol 2018;35(2):79–82. 10.4103/JOC.JOC_240_16 29643653 10.4103/JOC.JOC_240_16 PMC5885608

[16] CM K. Preparation of cells for microscopy using cytospin. Methods Enzymol 2013;533:235–40. 10.1016/B978-0-12-420067-8.00016-7 24182928 10.1016/B978-0-12-420067-8.00016-7

[17] Pooley RJ. Clinical hematology atlas. Arch Pathol Lab Med. 1999;123(11):1125 . 10539925

[18] Laboratory TJ. Mouse Phenome Database. Hematological survey of 11 inbred strains of mice. Available from: https://phenomejaxorg/.

[19] Rutella S, Rumi C, Lucia MB, Barberi T, Puggioni PL, Lai M, et al Induction of CD69 antigen on normal CD4+ and CD8+ lymphocyte subsets and its relationship with the phenotype of responding T-cells. Cytometry. 1999;38(3):95–101. . 10397327

[20] Obar JJ, Molloy MJ, Jellison ER, Stoklasek TA, Zhang W, Usherwood EJ, et al CD4+ T cell regulation of CD25 expression controls development of short-lived effector CD8+ T cells in primary and secondary responses. Proc Natl Acad Sci U S A. 2010;107(1):193–8. 10.1073/pnas.0909945107 ; PubMed Central PMCID: PMCPMC2806751. 19966302 10.1073/pnas.0909945107 PMC2806751

[21] Schlub TE, Badovinac VP, Sabel JT, Harty JT, Davenport MP. Predicting CD62L expression during the CD8+ T-cell response in vivo. Immunol Cell Biol. 2010;88(2):157–64. 10.1038/icb.2009.80 ; PubMed Central PMCID: PMCPMC2824781. 19859082 10.1038/icb.2009.80 PMC2824781

[22] van den Broek T, Borghans JAM, van Wijk F. The full spectrum of human naive T cells. Nat Rev Immunol. 2018 10.1038/s41577-018-0001-y . 29520044 10.1038/s41577-018-0001-y

[23] Posselt AM, Vincenti F, Bedolli M, Lantz M, Roberts JP, Hirose R. CD69 expression on peripheral CD8 T cells correlates with acute rejection in renal transplant recipients. Transplantation. 2003;76(1):190–5. 10.1097/01.TP.0000073614.29680.A8 . 12865808 10.1097/01.TP.0000073614.29680.A8

[24] Backer RA, Helbig C, Gentek R, Kent A, Laidlaw BJ, Dominguez CX, et al A central role for Notch in effector CD8(+) T cell differentiation. Nat Immunol. 2014;15(12):1143–51. 10.1038/ni.3027 ; PubMed Central PMCID: PMC4232996. 25344724 10.1038/ni.3027 PMC4232996

[25] Collin M, McGovern N, Haniffa M. Human dendritic cell subsets. Immunology. 2013 9; 140(1):22–30. 10.1111/imm.12117 ; PubMed Central PMCID: PMC3809702. 23621371 10.1111/imm.12117 PMC3809702

