# Supplementary figures and images for: A method for manual and automated multiplex RNAscope in situ hybridization and immunocytochemistry on cytospin samples
Source: PLoS One. 2018 Nov 20;13(11):e0207619. doi: 10.1371/journal.pone.0207619 (PMC6245747; doi:10.1371/journal.pone.0207619)

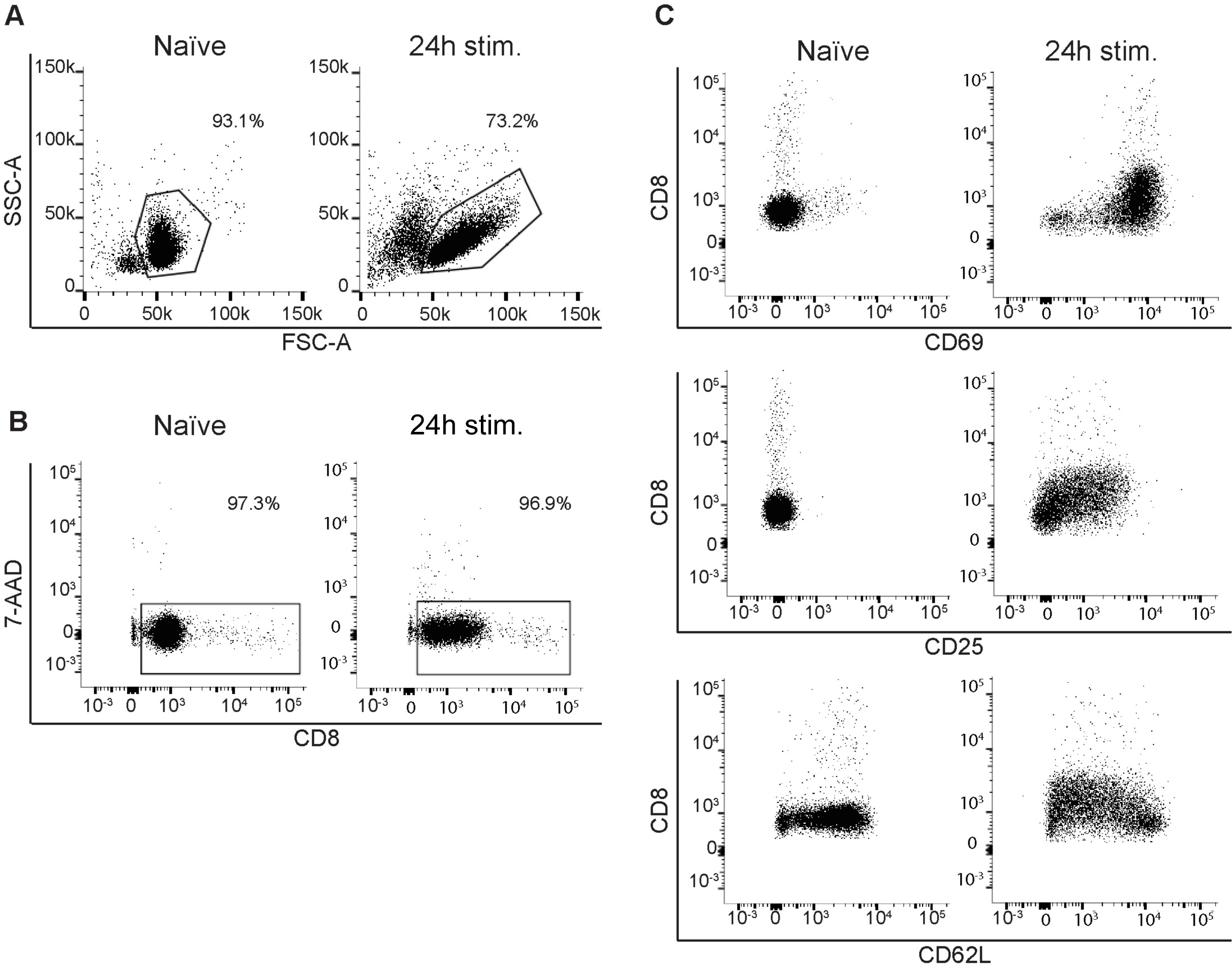

Supplement: S1 Fig — Representative FACS analysis of purified lymphocytes. (A) Lymphocytes were first identified in forward (FSC-A) and side scatter (SSC-A) dot plot to exclude debris, percentage represents gated fraction of total cells. (B) Of the previous population, only living (7-AAD negative) CD8+ T cells (CD8 positive) were considered for marker analysis. Please note that about 97% of purified living cells were CD8+. (C) The previous CD8+ population was analyzed for expression of CD69, CD25 and CD62L to access stimulation efficacy. (TIF) [file pone.0207619.s002.tif]

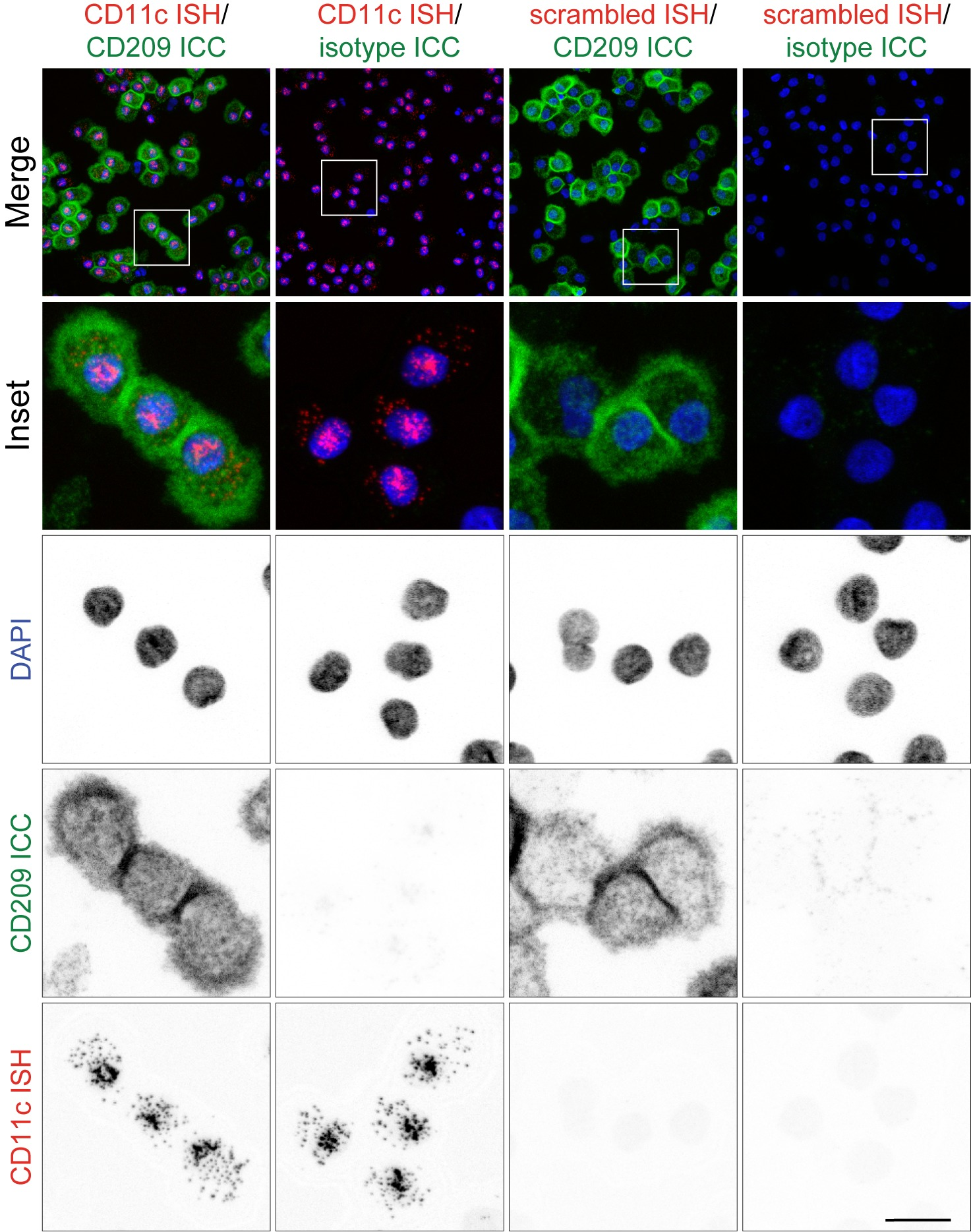

Supplement: S2 Fig — Representative images for dual ISH/ICC on human monocyte-derived dendritic cells. First row shows populations, second row is the zoomed in inset shown in the population images (white square). For inset images, split channels for each detector are shown in grey tone images to help visualization (third, fourth and fifth row). (TIF) [file pone.0207619.s003.tif]

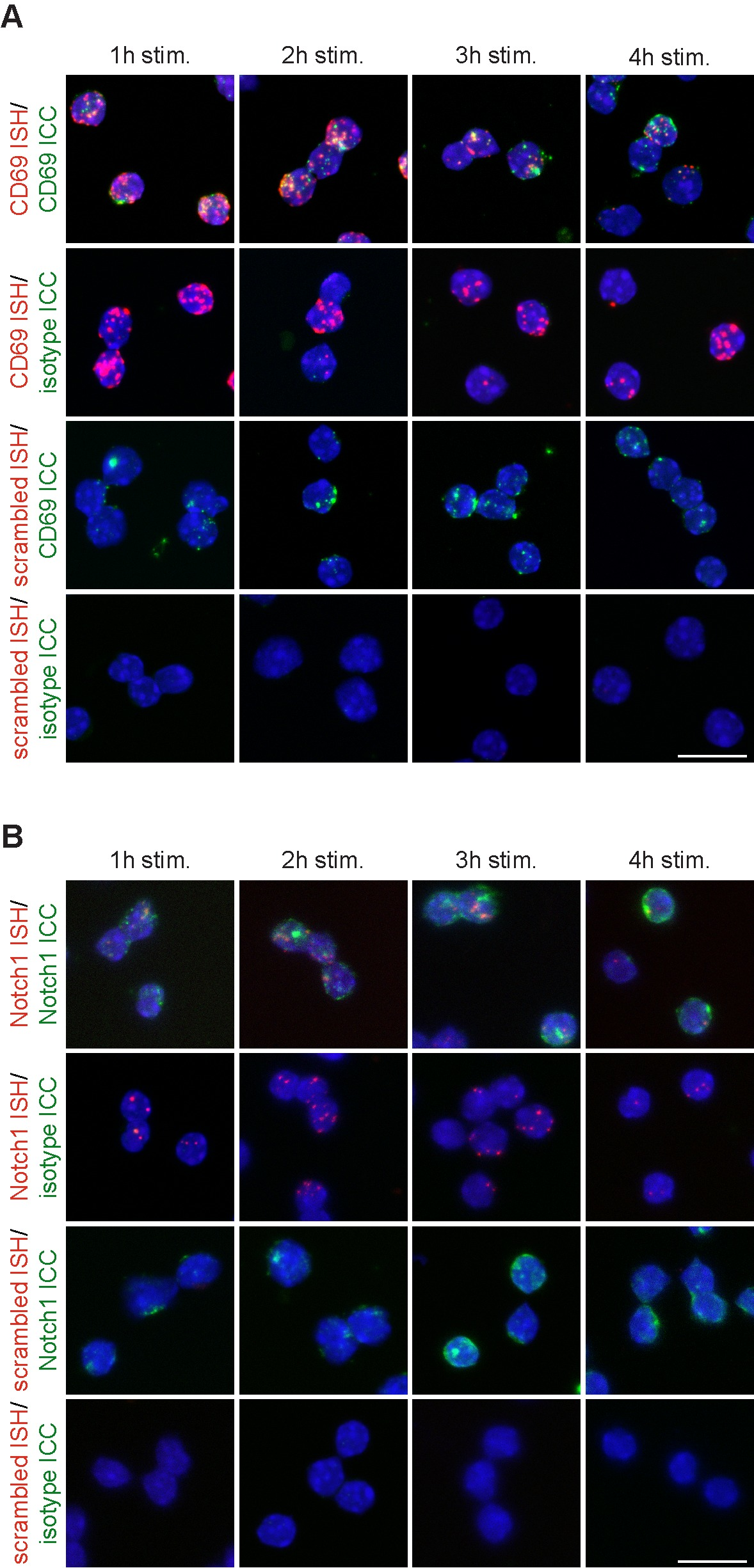

Supplement: S3 Fig — While only dual positive images are shown in Fig 4 for simplicity, here are shown all the controls performed. (A) Detection of CD69. (B) Detection of Notch1. In both panels: first row shows dual positive staining for ISH/ICC, second row shows ISH-only control in which specific probe was used in combination with an isotype for ICC. Images in the third row shows an ICC-only control, i.e. scrambled ISH probe combined with isotype antibody. The fourth row shows double negative control (scrambled sequence probe and isotype). (TIF) [file pone.0207619.s004.tif]

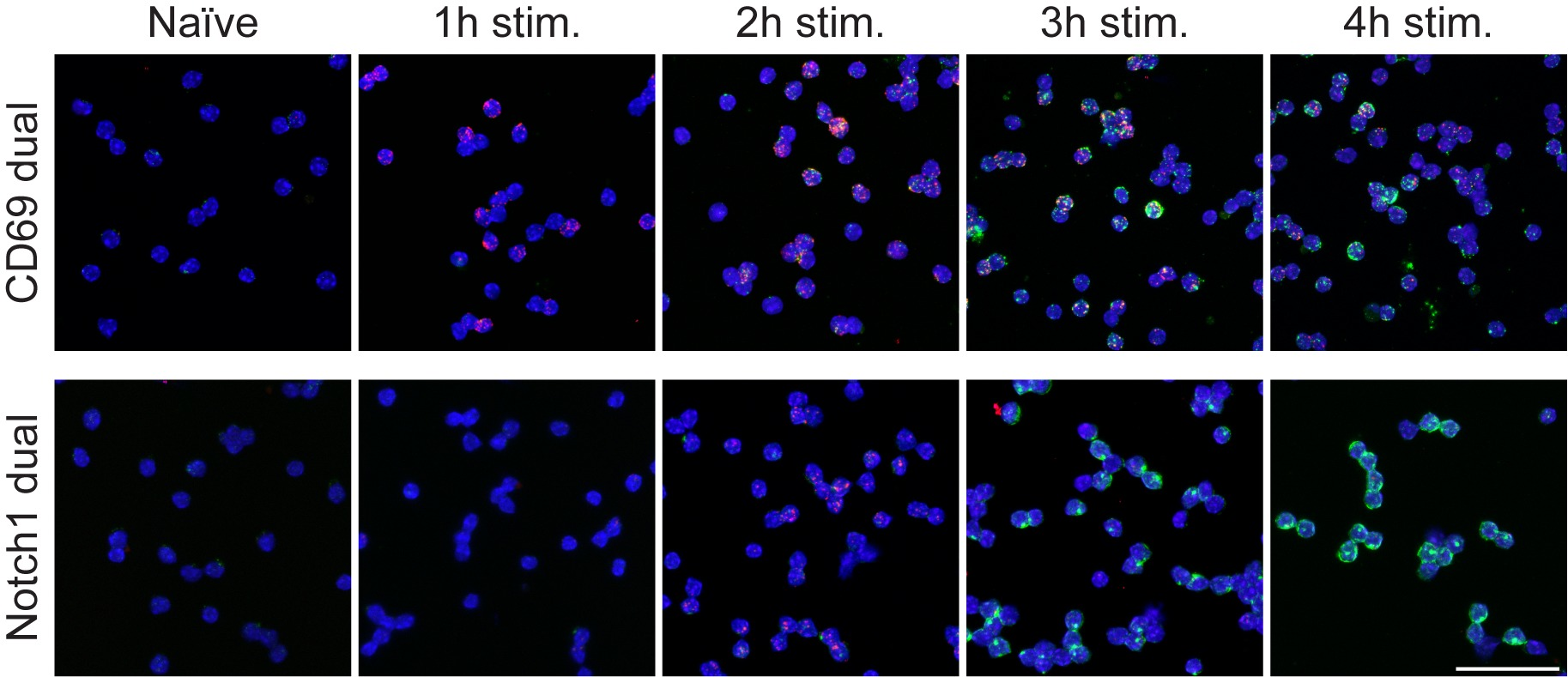

Supplement: S4 Fig — Cell populations from which insets shown in Fig 4 were taken. Top row shown dual ISH/ICC for CD69, bottom row shows dual ISH/ICC for Notch1. (TIF) [file pone.0207619.s005.tif]

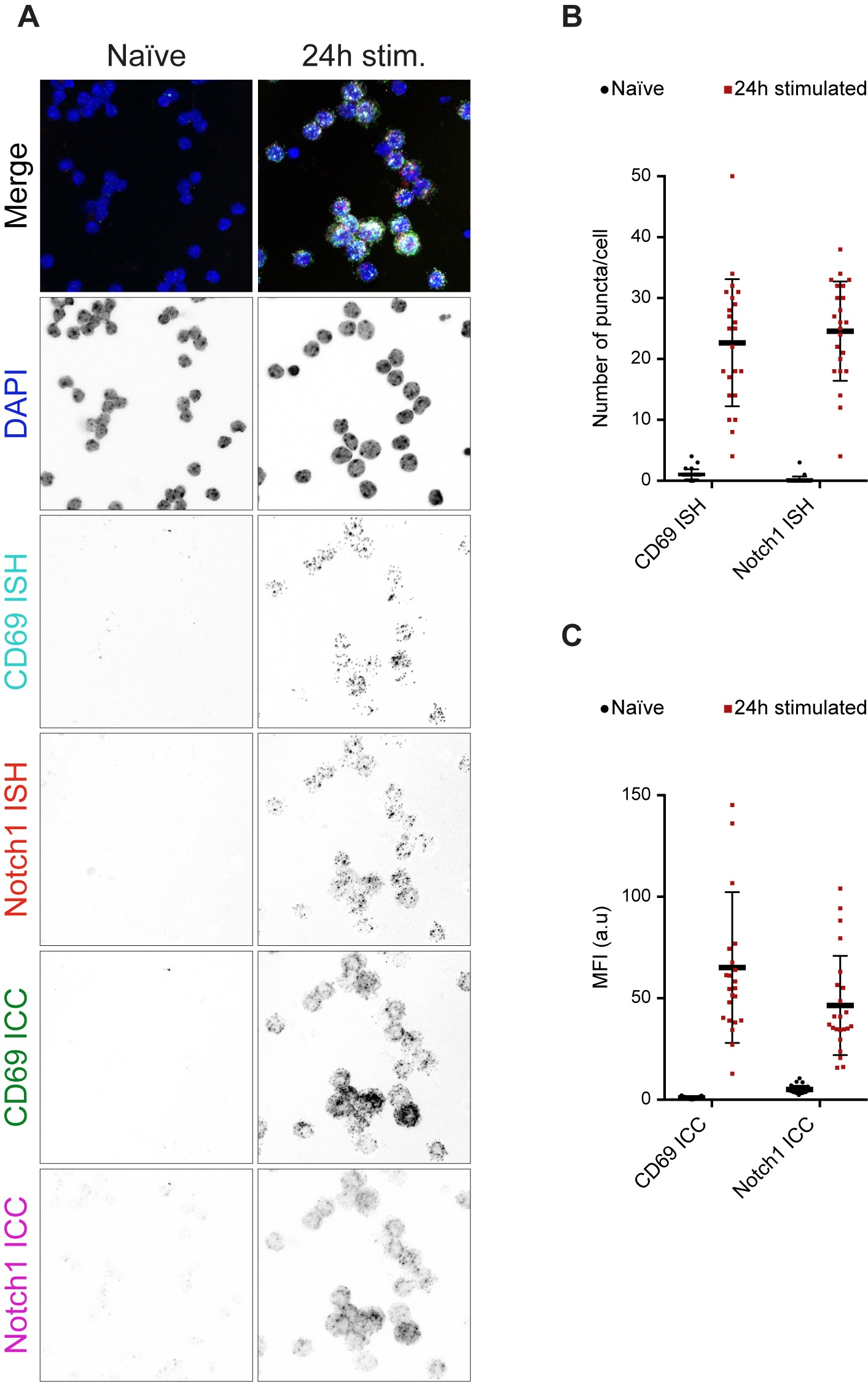

Supplement: S5 Fig — Quantification of signals for ISH and ICC for multiplex experiment. (TIF) [file pone.0207619.s006.tif]
